# Tuning WNT-β-catenin signaling via BCL9 proteins for targeting colorectal cancer cells

**DOI:** 10.1016/j.ebiom.2015.11.033

**Published:** 2015-11-19

**Authors:** Jean-François Beaulieu

**Affiliations:** Laboratory of Intestinal Physiopathology, Department of Anatomy & Cell Biology, Université de Sherbrooke, Sherbrooke, QC, Canada, J1H 5N4

The canonical WNT signaling pathway is ultimately involved in the regulation of cytoplasmic levels of free β-catenin. When inactive, the β-catenin not incorporated in adherent junctions is captured by the adenomatous polyposis coli (APC)-based protein complex, phosphorylated and then processed for degradation by the proteasome. Activation by WNT ligands such as those found in the intestinal stem cell niche located in the lower crypts prevents β-catenin ubiquitination allowing its accumulation in the cytoplasm and shuttling to the nucleus where it associates with the DNA-binding proteins of the lymphoid enhancer-binding factor/T-cell factor (TCF) family to transactivate specific gene expression such as *MYC*, *CCLD1* and other genes that drive cell proliferation and stemness (Niehrs 2012). Colorectal cancer (CRC) cells frequently display a constitutively active WNT–β-catenin signaling pathway as a consequence of mutations in *APC* or other genes that encode the APC-based protein destruction complex or β-catenin itself, which allows β-catenin to accumulate in the nucleus and contribute to cellular transformation (Barker and Clevers, 2006, Krausova and Korinek, 2014). There are many factors that interact and modulate the WNT cascade in both normal and transformed cells. As depicted in seminal reviews (Cruciat and Niehrs, 2013, de Lau et al., 2014), WNT signaling is strictly controlled at the ligand–receptor level by a series of inhibitors and activators that regulate signal strength. Furthermore, WNT signaling is also modulated at the transcriptional level by a series of β-catenin-interacting co-factors such as cyclic AMP response element-binding protein and B-cell lymphoma 9 (BCL9 and BCL9L) which can strengthen the activity (Holland et al. 2013). While many of these modulators of the WNT pathway represent potential targets for cancer therapeutics, their disruption can also lead to alterations in the WNT signaling pathway of healthy tissues, a difficulty that has to be taken into consideration in the design of pre-clinical and, eventually, clinical studies (Barker and Clevers 2006).

In this context, it is interesting to note that the impact of the cellular response associated with the β-catenin co-factor BCL9/9L may be context-dependent. Indeed, in the mouse, ablation of Bcl9/9l in the intestinal epithelium abrogates the expression of genes related to epithelial–mesenchymal transformation (EMT) and stemness in chemically induced colorectal tumors suggesting that the traits associated with tumor invasion and metastasis via Bcl9/9l appear to be dispensable for normal intestinal epithelial homeostasis (Deka et al. 2010).

In this issue of EBioMedicine, Moor et al. have further investigated the importance of BCL9/9L on WNT signaling in CRC cells (Moor et al. 2015). They first investigated a second CRC mouse model driven by loss of *Apc* and oncogenic *Kras* to complement their initial observations performed on the chemically induced mouse CRC model mentioned above (Deka et al. 2010) confirming that ablation of *Bcl9*/*9l* modulates similar alterations of gene expression in both types of tumors with 359 down- and 107 up-regulated common differentially expressed genes. Analysis of the up- and down-regulated genes revealed a negative enrichment of genes related to intestinal WNT targets, stemness and EMT while differentiation-related genes were positively enriched. Interestingly, a search for human homologs allowed the characterization of a BCL9/9L-KO signature of 378 genes that was then used to probe public human CRC databases revealing that hazards of relapse and death are both significantly reduced in patients displaying BCL9/9L-KO-like tumor profiles. Then, using the organoid/mini-gut culture system consisting of expanding epithelial stem cells isolated from the intact intestine into three-dimensional structures (Sato and Clevers 2013), they confirmed that ablation of *Bcl9*/*9l* improves morphological differentiation and generates a BCL9/9L KO-like signature without altering proliferation in vitro. Interestingly, subcutaneous grafting of the same organoids in immuno-compromised mice resulted in tumor regression in allografts lacking *Bcl9*/*9l* while the wild-type organoids grew exponentially, consistent with their *Apc −*/*−* and mutated *Kras* background. Taken together, these results suggest that CRC cells are very dependent on functional BCL9 proteins for promoting tumor progression via stimulation of stemness and EMT traits and preventing differentiation, a pathway not apparently essential for the maintenance of normal intestinal epithelial homeostasis. The discovery that patients with tumors displaying gene expression profiles similar to the BCL9/9L-KO signature have a better prognosis than those with a BCL9/9L wild-type phenotype is quite relevant to these findings. While this points out the interest of the β-catenin–BCL9/9L complex as a potential therapeutic target for CRC, it also raises the need to validate, in the end, the direct implication of BCL9/9L in the generation of the BCL9/9L-KO signature in human cells, considering the fact that *BCL9*/*9L* have been found to be expressed in all tumor samples. Another key aspect that remains to be investigated pertains to the possibly distinct implications of BCL9 and BCL9L. The findings from human CRC database analyses that stemness traits appear to correlate much more closely with BCL9L than BCL9 is noteworthy particularly in the context where in the mouse, BCL9L over-expression appears to induce intestinal tumorigenesis and promote expression of genes involved in EMT (Brembeck et al. 2011).

In summary, the results by Moor et al. show that inhibiting the BCL9/9L interaction with β-catenin leads to diminished gene expression associated with stemness and EMT traits and promotes differentiation in CRC tumors (in the context as shown previously (Deka et al. 2010) that it remains relatively well tolerated in the normal intestinal epithelium). Taken in conjunction with the fact that the BCL9/9L-KO signature in CRC tumors is associated with a better prognosis, the study points out that the BCL9/9L–β-catenin complex represents a promising potential therapeutic target for treating CRC.

## Disclosure

The author declares no conflicts of interest.
